# Temporal Electroencephalography Traits Dissociating Tactile Information and Cross-Modal Congruence Effects

**DOI:** 10.3390/s24010045

**Published:** 2023-12-21

**Authors:** Yusuke Ozawa, Natsue Yoshimura

**Affiliations:** 1 School of Engineering, Tokyo Institute of Technology, Yokohama 226-8503, Japan; ozawa.y.ai@m.titech.ac.jp; 2 School of Computing, Tokyo Institute of Technology, Yokohama 226-8503, Japan

**Keywords:** EEG, event-related potentials, object recognition, somatosensory evoked potentials, classification analysis

## Abstract

To explore whether temporal electroencephalography (EEG) traits can dissociate the physical properties of touching objects and the congruence effects of cross-modal stimuli, we applied a machine learning approach to two major temporal domain EEG traits, event-related potential (ERP) and somatosensory evoked potential (SEP), for each anatomical brain region. During a task in which participants had to identify one of two material surfaces as a tactile stimulus, a photo image that matched (‘congruent’) or mismatched (‘incongruent’) the material they were touching was given as a visual stimulus. Electrical stimulation was applied to the median nerve of the right wrist to evoke SEP while the participants touched the material. The classification accuracies using ERP extracted in reference to the tactile/visual stimulus onsets were significantly higher than chance levels in several regions in both congruent and incongruent conditions, whereas SEP extracted in reference to the electrical stimulus onsets resulted in no significant classification accuracies. Further analysis based on current source signals estimated using EEG revealed brain regions showing significant accuracy across conditions, suggesting that tactile-based object recognition information is encoded in the temporal domain EEG trait and broader brain regions, including the premotor, parietal, and somatosensory areas.

## 1. Introduction

Humans recognize objects through both their tactile and visual senses in the real world, and for the brain, it is crucial to properly perceive and integrate inputs from multiple sensory systems to perform object recognition. In particular, the somatosensory and visual systems are known to share shape features and complement each other’s inputs in the object recognition process [1]. Several studies have demonstrated that the perception of tactile information is influenced by the degree of congruence between modalities in integrating tactile and visual information [2,3,4,5], suggesting that sensory inputs from visual and tactile receptors may be flexibly modulated in the central nervous system in response to ongoing behavioral associations.

The integration of tactile and visual information in object recognition must occur at a single stage or multiple stages in the stream of visual and tactile sensory processing from the sensory receptors, early and late subcortical relay neurons, or within the cerebral cortex. To understand and elucidate this integration mechanism, it is common to present multimodal sensory stimuli and use approaches in which brain regions and temporal patterns of brain activity contribute to object recognition [6,7].

In animals, electrophysiological methods have shown that multisensory and visual tactile neurons are present in area 7b [8], the ventral intraparietal area (VIP) [9,10], the premotor cortex [11,12], and the superior temporal sulcus [13]. In humans, functional magnetic resonance imaging (fMRI) has indicated the involvement of the lateral occipital (LO) region in shape representation produced by tactile and visual sensations [14] and in the discrimination between visual and tactile sensations [15]. In addition, the involvement of the frontal-parietal-cerebellar and premotor regions has been identified in a few studies [16,17,18,19]. These studies suggest that visual and tactile integration occur in multiple areas. However, the brain region that contributes to the integration of multimodal information in the early temporal stage has not been fully investigated, as electrophysiological methods cannot cover the entire brain and fMRI does not have high temporal resolution.

To address this issue, several studies have used electroencephalography (EEG), which can be employed to record signals from the whole brain with high temporal resolution [20,21,22]. These studies reported the involvement of multiple regions other than M1 and S1. However, most of these studies used frequency-domain traits, such as event-related spectral perturbation (ERSP), and it is unclear whether the temporal domain traits of EEG, such as event-related potential (ERP) and sensory evoked potential (SEP), also represent neural activity related to multisensory integration when touching an object.

ERP is a form of potential that can be recorded using electrodes placed on the scalp in response to a recognitional or attentional event. The latency of ERP is around 300 ms or 1500 ms, and its amplitude is known to vary according to the semantic congruency of contextual understanding and visual attention [23,24]. On the other hand, SEP is a type of potential that can be observed in the pathway from the central nervous system to the brain according to electrical nerve stimulation of the limbs. The latency of SEP is faster than that of ERP, amounting to 20 or 25 ms. SEP reflects the effect of the early stage of sensory processing, and its amplitude is known to be modulated during voluntary movements and when self-identification is altered by the congruency of tactile and visual stimuli [25,26,27,28,29,30]. Thus, as amplitudes of both ERP and SEP are modulated depending on tasks, it is interesting to investigate whether they are also affected by visual and tactile congruency in object recognition. In addition, investigating brain regional differences in the way such modulation occurs would contribute to understanding sensory processing mechanisms.

Therefore, in this study, we examined whether ERP or SEP possess information that represents tactile-based object recognition and examined its cross-modal congruency effects. The participants were presented with cross-modal stimuli consisting of tactile and visual stimuli, and their EEG signals were recorded while they were judging what object they were touching by directing their attention to either tactile or visual stimuli. ERP and SEP in anatomical brain regions were calculated using the variational Bayesian multimodal encephalography method [31] as an EEG source localization method, and the machine learning-based classification accuracies of touched materials using ERP and SEP were compared under conditions of the congruence or incongruence of visual and tactile stimuli using a sparse logistic regression (SLR) algorithm [32]. If there are brain regions able to discriminate material differences with significantly high accuracy under cross-modal stimulation conditions, this would suggest that both frequency and temporal-domain traits represent neural processes of tactile-based object recognition and cross-modal stimulus congruency effects.

## 2. Materials and Methods

### 2.1. Participants

Twelve healthy participants (eleven males, with a mean age ± standard deviation of 23.58 ± 1.56 years) with normal or corrected-to-normal vision participated in the experiment reported herein. The details of these participants are summarized in Table 1. This study was conducted in accordance with the protocol approved by the Ethics Committee of the Tokyo Institute of Technology (ethics number: 2015062). All participants provided written informed consent in accordance with the Declaration of Helsinki.

### 2.2. Experimental Paradigm

**Figure 1:**
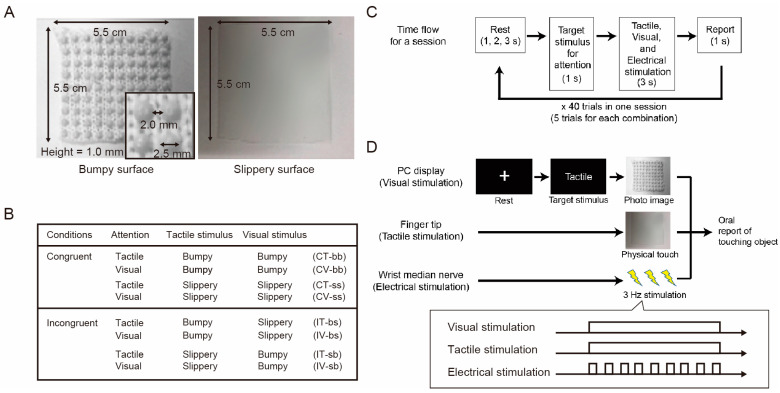
Experimental design. (**A**) Characteristics of two materials with different degrees of surface smoothness (i.e., bumpy and slippery) used in the experiment. For tactile stimuli, one of the physical materials was presented to the right index finger, and for visual stimuli, one of the photo images of the materials was presented on a PC display. (**B**) A table summarizing eight combinations of attention, tactile, and visual stimuli in the two experimental conditions, congruent and incongruent. (**C**) Time flow for a session. One trial consisted of four periods that lasted 6–8 s in total depending on the lengths of the rest period. In the second period, one of the two target stimuli, tactile or visual, was shown on the screen for 1 s so that the participants would pay attention to the instructed stimulus in the next period. In the third period of 3 s, a photo image of the two materials was presented on the screen, and one of the physical materials was presented to the corresponding participant’s index finger. Electrical stimulation was also applied to the right wrist median nerve throughout the period. In the fourth period, the participants verbally reported which material they were touching within 1 s. One session included 40 trials consisting of 5 trials for eight individual combinations in random order. One participant engaged in three sessions in total. (**D**) An example trial of stimulus presentation. This example shows an incongruent condition in which the bumpy surface is presented as a visual stimulus and the slippery surface is presented as a tactile stimulus during tactile attention. When visual and tactile stimuli were presented, electrical stimulation was also presented to evoke SEP. During the stimulus presentation time, visual and tactile stimulation were presented continuously but electrical stimulation was presented discretely.

We developed an experimental system in which two sheet-like materials with different surface roughness values (bumpy and slippery materials, as shown in Figure 1A) were presented as tactile stimuli, and photographic images of the surfaces of the two materials were used as visual stimuli so that the participants could touch the materials while viewing the images. In this experiment, these two simple materials were used because the purpose of this study was to determine the effect of congruency between visual and tactile stimuli on ERP or SEP, not to investigate the effects of the different materials. The bumpy surface consisted of hemispheres with a diameter of 2.5 mm and a height of 1.0 mm, lined up every 2.0 mm, and made of polyester and cotton. The slippery surface was completely flat and made of plastic (Figure 1A).

To provide a cross-modal stimulus presentation, we designed a condition in which the presented materials were congruent or incongruent with the tactile and visual stimuli (Figure 1B). The tactile stimuli (actual materials) were manually presented by an experimenter at the same time as the visual stimuli (photo images) were presented on the PC screen using MATLAB software (version R2020b) to ensure that the participants could not see their own hands or the tactile stimuli (i.e., materials) and could touch the materials with their fingertips. In MATLAB, we used plugins including Wavelet toolbox, Statistics and Machine Learning Toolbox, Signal Processing Toolbox, and Instrument Control Toolbox.

To eliminate the effects of differences in the stimuli to which each participant paid attention, we prepared four combinations, congruent with tactile attention (CT), congruent with visual attention (CV), incongruent with tactile attention (IT), and incongruent with visual attention (IV), for each material. In each combination, the participants directed their attention to either tactile or visual stimulus (Figure 1B) according to an instruction given before each trial. In the case of tactile attention, the participants tried to pay attention to their fingertips instead of the material shown on the display. On the other hand, in the case of visual attention, they tried to pay attention to what they saw on the display instead of the material presented to their fingertips. In these eight combinations, the participants were instructed to judge which material they thought they were touching while their EEG signals were recorded. As simple and easy-to-recognize materials were used in this study, complex recognitional processing was not required.

The detailed procedure is as follows. The participants sat in a comfortable chair in front of a monitor placed on a desk with an armrest and adjusted the positions of the seat and armrest to suit their comfort level. The right arm of each subject was placed on an armrest. The participants listened to white noise using earphones to block out auditory information.

Figure 1C shows the flowchart of one session. Each trial consisted of the following four steps: In the first step, during the rest period, a white fixation cross appeared on the screen, and the participants gazed at the cross without engaging in any other actions. The length of the period was randomly selected as 1, 2, or 3 s. In the second step, one of the target stimuli to which the participants were instructed to pay attention was indicated by the English words “Tactile” or “Visual” for 1 s. In the third step, a photographic image of either material was displayed on the screen for 3 s, and either material was passively and constantly presented to the fingertips of the participant’s right hand. To evoke SEP, electrical stimulation was simultaneously applied to the median nerve of the right wrist (Sections II. F. SEP). In the fourth step of 1 s, the participants verbally indicated which material they touched by being aware of their fingertip sensations when the instruction in the second phase was “Tactile” or by being aware of the photo on the screen when the instruction was “Visual”. Each session consisted of 40 trials, with 5 trials for each combination presented randomly. The participants engaged in three sessions.

Figure 1D shows an example of the IT-sb condition (incongruent stimulation condition under tactile attention (tactile: Slippery; visual: Bumpy), see Figure 1B). First, the fixation cross was displayed in the rest period, and then the target stimulus for attention, Tactile, was shown for 1 s. Then, a photo image of the bumpy material was displayed, and the slippery surface material was presented to the participants’ fingertips. During this period, electrical stimuli were repeatedly presented to the right wrist at 3 Hz, but this process was not synchronized with the tactile and visual stimuli to avoid diverting attention away from the tactile or visual stimuli. The electrical stimuli were not synchronized with the visual and tactile stimuli in that they were presented discretely at 3 Hz, while the visual and tactile stimuli were presented continuously during the stimulus presentation period (Figure 1D).

### 2.3. Data acquisition and Preprocessing

**Figure 2:**
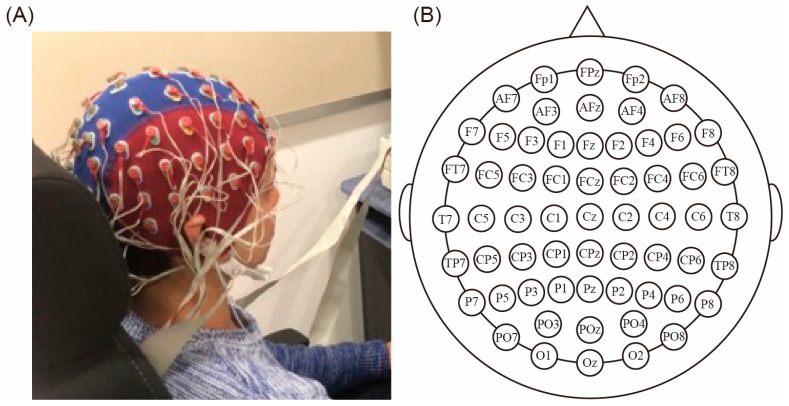
EEG settings. (**A**) The EEG setup. (**B**) Electrode placement based on the international 10–20 method.

Throughout the experiment, EEG signals were acquired from 64 electrodes using a Biosemi ActiveTwo system (Biosemi, Amsterdam, Netherlands) designed according to the extended international 10–20 system [33]. The EEG setup and the electrodes’ arrangement are shown in Figure 2A,B. The signals were sampled at 2048 Hz. The offline EEG analyses were performed using MATLAB (version R2020b). EEGLAB [34] was used for preprocessing. The EEG signals were down-sampled to 512 Hz, high-pass-filtered at 1 Hz [35], and processed using a function called cleanline (version 2.00) [36] to remove power supply noise. The Common Average Reference (CAR) [37] was applied as a reference. Tactile/visual and electrical stimulus timing periods were used for ERP and SEP calculations, respectively. For classification using ERP, each epoch was extracted from 1000 ms before to 3000 ms after the initiation of tactile/visual stimulation. For classification using SEP, each epoch was extracted from 100 ms before and after the electrical stimulation.

### 2.4. Cortical Current Source Estimation

To investigate the differences in accuracy in the brain regions between the conditions, we used cortical current source estimation. Various techniques can be used to estimate cortical current sources, such as EEG, magnetoencephalography (MEG), and functional magnetic resonance imaging (fMRI) [38]. Among these techniques, we used the variational Bayesian multimodal encephalography method, carried out via VBMEG toolbox version 3.0 (ATR Neural Information Analysis Laboratories, Kyoto, Japan) [31,39]. Cortical current source signals were calculated by applying an inverse filter to the EEG sensor signals. The inverse filter was estimated separately for each participant by conjointly considering the variance in baseline EEG signals, EEG sensor coordinates in the standard brain, and a standard brain model constructed from the standard MRI brain image. Cortical current source signals were used to calculate ERP and SEP.

### 2.5. ERP

ERPs were extracted based on the onset of visual or tactile stimulation. The number of ERPs for each of the eight conditions was 15 (i.e., five trials with three sessions). Because we performed 2-class material classification in the congruent and incongruent conditions by combining the eight condition datapoints, the total number of trials of one class (congruent or incongruent) was 60 (i.e., CT-bb, CV-bb, CT-ss, and CV-ss for the congruent class, and IT-bs, IV-bs, IT-sb, and IV-sb for the incongruent class). We identified a peak amplitude of N600.

### 2.6. SEP

The effects of the early stage of sensory processing were observed in reference to SEP. Therefore, the electrical stimulation employed to evoke SEP was presented using visual and tactile stimuli to investigate whether visual and tactile congruency affect the early stage of sensory processing. SEPs were computed with reference to the onset of electrical stimulation. Electrical stimulation was repeatedly applied to the right median nerve of the wrist (3 Hz, with a 0.2 ms pulse duration). The electrical stimuli were not synchronized with the visual and tactile stimuli to avoid distracting the participants from these stimuli. The stimulation was delivered as a square-wave pulse current using an SS-104J isolator (Nihon-Koden, Tokyo, Japan). In the experiment, the electrical stimulation current was determined for each participant at an intensity slightly lower than the motion threshold at which a finger twitch occurred. The total number of electrical stimuli was 135 for each condition. Thus, the total number of electrical stimuli was set to 540 for the congruent and incongruent conditions [40]. SEP was epoched from −100 ms to 100 ms in reference to onset of electrical stimulation. The peak amplitudes of the N20, P25, N33, P45, N57, and P65 components were identified.

### 2.7. Classification Analysis

We divided the 3 s task-period data into three temporal sections (0–1 s, 1–2 s, and 2–3 s) to examine the temporal effects of object recognition. A two-class classification of the two materials was performed for each congruent and incongruent condition and for each brain region. We used sparse logistic regression (SLR) [32] for classification, which combines linear regression with automatic relevance determination (ARD) to perform feature selection and train the model for classification. In SLR, weights that are irrelevant to the classification are automatically set to zero, leading to a sparse weight vector. This allows SLR to train high-dimensional classifiers without requiring advanced feature-dimension reduction and prevents overfitting. We used the SLR toolbox version 1.2.1. SLR was used because it can train high-dimensional classifiers without prior feature selection. In machine learning, problems such as overfitting, which is when the dimensionality of the input data is large in relation to the number of data, occur, but SLR can automatically reduce features that are not important for learning. In this study, the time-series signal itself was used as the input for the EEG analysis parameter. The number of time points was 512 (512 samples × 1 s) for ERP analysis, and there were 102 points (512 samples × 0.2 s) for SEP. For ERP classification, there were 54 training data and 6 test data. For SEP classification, there were 486 training data and 54 test data.

The cortical-current-source-based ERP or SEP within each brain region was used as the feature vector, and classification was performed for each brain region to investigate which brain regions contain enough information to discriminate information associated with congruency between visual and tactile sensation. Classification accuracy was calculated using ten-fold cross-validation. Classification performance was compared to accuracies for the same classifiers using shuffled labels to determine whether the classification performance using real-labeled data was higher than the chance level calculated using shuffle-labeled data. The brain regions used in the classification were the primary motor area (M1: area 4), the primary somatosensory areas (S1: areas 1, 2, 3a, and 3b), the secondary somatosensory area (S2: area 43), the paracentral lobule areas (PL: 5L, 5m, and 5mv), the supplementary motor region areas (SMA: 6ma, 6mp, SFL, and SCEF), the premotor areas (PM: 6a, 6d, FEF, 55b, PEF, 6v, and 6r), the anterior cingulate cortex (ACC: 33pr, 24dd, 24dv, p24pr, a24pr, p24, a24, p32pr, a32pr, d32, p32, s32, and area 25), the posterior cingulate cortex (PCC: d23ab, v23ab, 23c, 23d, and RSC), the supramarginal area (SMG: Ig, OP4, OP2-3, OP1, PFcm, RI, area 52, A1, MBelt, LBelt, PSL, and STV), the precuneus (PC: PCV, 7m), the inferior parietal lobule (IPL: PFop, PFt, PF, PFm, PGs, PGi, PGp, TPOJ1, TPOJ2, and TPOJ3), the intraparietal sulcus areas (IPS: IP0, IP1, IP2, IPS1, AIP, LIPd, LIPv, and MIP), the superior parietal lobule areas (SPL: 7PC, 7AL, 7AM, 7PL, 7PM, and VIP), the parieto-occipital sulcus areas (POS: POS1, POS2, DVT, and ProS), and occipital areas (Occ: V1, V2, V3, V4, V6, V8, PIT, FFC, VVC, VMV1, VMV2, VMV3, V6A, V3A, V3B, V3CD, LO1, LO2, LO3, V4t, MT, MST, FST, and PH) defined according to the human connectome project (HCP) [41,42,43,44,45].

### 2.8. Statistical Analysis

Before comparing the classification accuracies between the congruent and incongruent conditions, a normal distribution test was applied using the Shapiro-Wilk test [46]. Subsequently, we applied Mauchly’s test [47] to determine whether the data satisfied the assumption of sphericity. If they did not, the degrees of freedom were corrected using the Greenhouse-Geisser correction [48]. After these tests were applied, we calculated the regions of difference in the conditions using three-way repeated-measures analysis of variance (ANOVA) regarding brain regions, conditions (congruent and incongruent), and time periods (0–1 s, 1–2 s, and 2–3 s). Post hoc multiple comparisons between every group were executed using Tukey-Kramer correction [49] to identify brain regions in which the accuracy was higher than the chance level and in which differences appeared between congruent and incongruent conditions. Statistical significance was defined as *p* < 0.05.

## 3. Results

### 3.1. Classification Results for ERP and SEP

**Figure 3:**
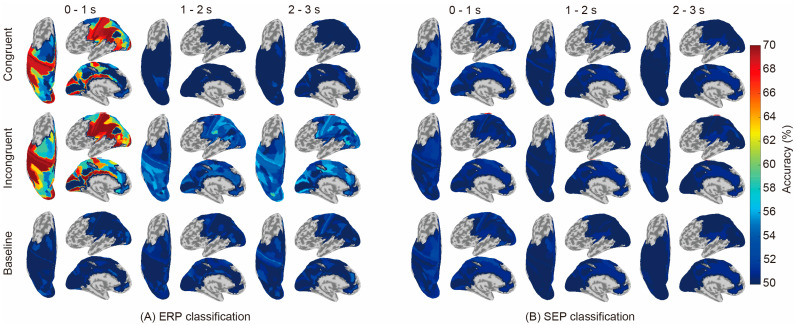
Classification accuracies between conditions using current-source-based ERP and SEP are displayed in each brain region. The classification was performed in the following areas: M1, S1, S2, PL, SMA, PM, ACC, PCC, SMG, PC, IPL, IPS, SPL, POS, and Occ. The three-second signal after the initiation of material presentation was divided into three periods (0–1 s, 1–2 s, and 2–3 s after the material was presented), and classification was performed for each time period. The classification accuracy of the shuffled labels (baseline) was used for comparison with congruent and incongruent conditions.

Figure 3 compares the classification results regarding ERP (A) and SEP (B). ERP showed significantly higher accuracy than the baseline (i.e., the chance level calculated using shuffled labels) in some regions and time periods, whereas SEP showed no significant difference from the baseline in all areas and conditions. The names of the brain areas and classification accuracies are shown in Tables S1 (congruent vs. baseline, 0–1 s), S2 (congruent vs. baseline, 1–2 s), S3 (congruent vs. baseline, 2–3 s), S4 (incongruent vs. baseline, 0–1 s), S5 (incongruent vs. baseline, 1–2 s), S6 (incongruent vs. baseline, 2–3 s), S7 (congruent vs. incongruent, 0–1 s), S8 (congruent vs. incongruent, 1–2 s), and S9 (congruent vs. incongruent, 2–3 s) in the Supplementary Materials.

### 3.2. Brain Regions Contributing to ERP-Based Classification

**Figure 4:**
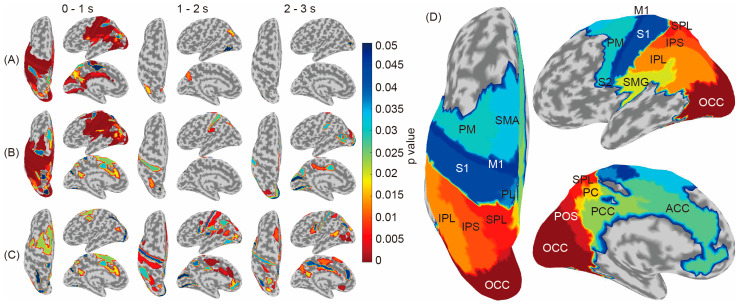
Brain regions showing significant accuracy differences compared to the chance level calculated using shuffled labels (congruent condition (**A**) and incongruent condition (**B**)) and significant differences between the congruent and incongruent conditions (**C**). The S1, PL, SMA, PM, ACC, PCC, SMG, PC, IPL, IPS, SPL, POS, and Occ areas showed a significant difference between the congruent and incongruent conditions. (**D**) Location of each brain region.

In Figure 4A,B, the brain regions showing significantly higher accuracies compared to the baseline are colored according to the *p*-values. The results indicated that the congruent condition (A) used wider-temporal-stage signals for classification; furthermore, the incongruent condition (B) required wider brain regions, including the SMA and the dorsal part of the PM, and wider temporal stages, including 1–2 and 2–3 s.

A comparison of the tables in the Supplementary Materials presenting significant brain regions in the congruent condition (Tables S1–S3) shows that the cingulate, SMG, parieto-occipital sulcus areas, and occipital area (ACC, SMG, POS, and Occ) contributed to the middle temporal stage (1–2 s) and the occipital area (Occ) contributed to the later temporal stages (2–3 s) in the incongruent condition (Tables S4–S6); that the sensory to parietal areas (S1, IPL, and IPS) contributed to the middle temporal stage (1–2 s); and that the cingulate to occipital areas (ACC, PCC, IPL, POS, and Occ) contributed to the later temporal stages (2–3 s).

Contrarily, Figure 4C and Tables S7–S9 in the Supplementary Materials, which show significantly different brain regions between the congruent and incongruent conditions (red), indicate that M1 did not represent information differences between the congruent and incongruent conditions and more complicated regional shifts from PM–oriented areas (0–1 s) to cingulate (1–2 s) and occipital-oriented areas (2–3 s). The names of each brain region are shown in Figure 4D.

## 4. Discussion

In this study, we performed classification analysis for each brain region using the ERP and SEP of EEG cortical current source signals as inputs to investigate whether temporal-domain EEG traits represent neural activity relating to the recognition of the physical properties of objects. Only ERPs achieved significantly higher accuracy than the baseline in both conditions, where the tactile and visual stimuli were either congruent or incongruent (Figure 3). Considering that the results regarding brain regions showing significantly high accuracy are consistent with results from previous studies using frequency-domain traits, it is suggested that ERPs represent neural activity during tactile-based object recognition and their differences according to cross-modal congruence effects. In addition, Figure 4 further reveals that the later components of the ERP (1–2 s and 2–3 s) represent the neural activity corresponding to object recognition in the incongruent condition.

Whether the congruency of visual and tactile stimuli also affect N600 has not been investigated. Previous studies have reported that the modulation of N600, the late component of ERP, occurred via semantic congruency. Stevens et al. presented semantically congruent and incongruent visual stimuli under varying distance and gaze conditions to investigate neural processing during the development of a contextual understanding of “here” and “there”. They showed that a larger N600 amplitude was induced in the left temporal region during the congruent task compared to that in the incongruent task [23]. Wang et al. presented several images consisting of similes, metaphors, and analogies, which corresponded to congruent, related, and incongruent meanings, respectively, and asked participants to categorize them during ERP measurement. They showed that a larger N600 amplitude was induced in the left frontal region during the incongruent task than during the congruent task [50]. Thus, whether the ERP amplitude was larger in the congruent or incongruent conditions was dependent on the type of task; but in the left hemisphere, the difference between the conditions was observed to depend on the semantic congruency or incongruency of the stimuli. In contrast to these studies, the present study suggests that when focusing on the late stages of sensory processing, differences in the effects of congruency between visual and tactile information can be found in multiple brain regions in the left hemisphere (i.e., corresponding to the contralateral part of the touching finger). This result suggests that the congruency of visual and tactile stimuli also affects N600.

The regions involved in discriminating congruency between visual and tactile stimuli were M1, S1, S2, PL, SMA, PM, ACC, PCC, SMG, PC, IPL, IPS, SPL, POS, and Occ, and these regions have been confirmed to be affected by congruency in previous studies using EEG frequency-domain traits. Krebber et al. investigated the effects of visual and tactile motion congruency on the brain using ERSP and confirmed the involvement of the sensorimotor areas (S1 and M1) [20]. Wang et al. investigated the effects of pattern matching between visual and tactile stimuli using phase-amplitude coupling and found that M1, PL, SPL, IPS, the secondary somatosensory cortex (S2), and the supramarginal gyrus (SMG) were involved [21]. Goschi et al. investigated ERSP analyzed using EEG during a target detection task wherein stimuli were presented using congruent and incongruent visual and tactile stimuli and showed that PM, SMA, and SMG were involved [22]. Gentile et al. presented participants with stimuli that varied the spatiotemporal congruency between visual and tactile stimuli and used fMRI to examine the neural activity that occurred during the presentation of the stimuli. Activation occurred in the PL and PM regions [18]. In addition, Limanowski et al. used fMRI to investigate the effect of congruency between visual and proprioceptive information regarding arm position and found changes in the IPL and PM [19]. Thus, our results suggest that the areas where the influence of visual and tactile congruency was confirmed in the frequency domain could also represent information in ERP as a temporal-domain trait. The congruency between visual and tactile stimuli was shown to involve not only early processing stages such as S1 but also higher-order regions. The identification of these regions achieved in this study might contribute to our understanding of the sensory processing circuits involving vision and touch. It is expected that network analysis could be applied to the regions suggested in the present results to investigate signal flow.

There were no significant differences in classification using the SEP components (Figure 3). The reason for this insignificance may be due to the difference in neural signaling between SEP and ERP. Since the SEP components focused on in this study have been reported to reflect the activation of somatosensory areas [25,30,51,52,53] and information from the sensory receptor through subcortical relay neurons to areas 1, 3b, or S1, SEP reflects the early stage of sensory processing within 100 ms after the stimulus has been applied, which might indicate that visual information has no effect on the tactile information itself. On the other hand, ERP reflects the late stage of sensory processing around 300 ms or 1500 ms after the stimulus had been triggered and includes the influence of higher-order perceptional information rather than the tactile information itself. Therefore, the fact that it was not significant in SEP and was significant in ERP suggests that it is likely that the discrepancy between visual and tactile information modulates intercortical signaling with respect to performing higher-order perceptional processing rather than signaling from sensory receptors to subcortical relay neurons. In addition, studies using oscillation have suggested the involvement of β band activity in tasks involving the detection of tactile stimuli regardless of visual stimulation [22] and of μ band activity in tasks involving the evaluation of the pleasantness of tactile stimuli within 1 s [54]. Thus, in studies using low-frequency oscillation such as β and μ bands, there is a difference in ERSP between conditions during the first second after stimulus presentation. Hence, the high classification accuracy during the first second after stimulus presentation observed in the present ERP analysis suggests that important information for tactile might be present during this period.

The modulation of the early components of SEPs has been reported in several studies based on slightly different paradigms, such as using visual stimuli of a person being exposed to tactile stimulation. These kinds of paradigms might have self-identification-related rather than sensory influences. The following studies have examined the spatial congruence of visual and tactile information: Deschrijver et al. stimulated the index and middle fingers using tactile stimuli while also stimulating them using visual stimuli, which were congruent or incongruent with which finger to stimulate, investigating whether the congruency of these stimuli-modulated SEP. They showed that the P50 amplitude was larger when the stimuli were congruent rather than incongruent [26]. In addition, Cardini et al. stimulated the middle and ring fingers and presented a finger that was stimulated as a visual stimulus in conditions that were congruent or incongruent to the actual tactile stimulus. The authors showed that the somatosensory suppression index (SSI) at P100 was higher in the congruent condition, indicating that body-congruent sensory information is important for organizing somatosensory perception [27]. Studies have investigated the congruence of visual stimuli with respect to how tactile stimuli are presented and followed. Aspell et al. presented a virtual body being stroked on its back as a tactile stimulus and a congruent or incongruent stroking video as a visual stimulus while measuring SEP. They showed that the P40 of the electrodes near the sensory area was larger in the incongruent condition [28]. Sakamoto et al. stroked a participant’s hand and a fake hand with tactile stimuli while showing them a fake hand. They measured SEP under conditions of congruent and incongruent stroke timing and position and showed that the N20/P25 component was attenuated in the congruent condition [29]. These studies show that SEP is affected by the intersection of vision and touch in self-perception. In this study, the SEP may not have changed because the actual object was presented and self-perception was not involved.

In conclusion, cross-modal effects of visual and tactile stimuli of touching an object were only observed in ERPs. The brain regions where modulation of ERP was observed were consistent with regions suggested in previous studies using frequency analysis. This result suggests that the influence of tactile and visual congruency also appears in the time-domain trait. Furthermore, a few areas showed differences in stimulus congruency, even in the later temporal period, of 1 to 3 s after the object’s presentation. These results add further insight to cross-modal studies of visual and tactile stimuli.

However, considering the following limitations and future possibilities would further enhance the findings for future applications.

(1) The materials used in this study were two easily distinguishable materials with completely different degrees of surface roughness. It is expected that improving the similarity of the materials would decrease discrimination accuracy. Therefore, it would be interesting to investigate the degree to which stimulus similarities can be discriminated. For example, it may be possible to seek the limits that could provide greater perception while giving the user a more realistic experience using VR technology. To clarify this possibility, it is necessary to use materials with similar characteristics of surface roughness in future studies.
(2) In addition, it would be useful to conduct an experiment with additional tasks for either visual or tactile stimuli and no tactile or visual stimuli (i.e., only electrical stimulation) and then analyze the results because it may be possible to dissociate responses that may be associated with sensory integration by subtracting brain responses ranging from unimodal to multimodal conditions [55,56].
(3) In this study, it was expected that the congruency effect between visual and tactile information would appear in the process of perceptual processing because the participants judged what object they were touching. Therefore, it would be interesting to investigate where changes in brain activity due to congruency effects appear. To this end, network analysis may be useful to capture the signal flow and brain regions that contribute to the effect.

## Figures and Tables

**Table 1:** Patients’ information.

Subject	Sex	Age	Dominant Hand
1	Male	23	Right
2	Male	22	Right
3	Male	22	Right
4	Male	23	Right
5	Male	24	Right
6	Male	22	Right
7	Male	23	Right
8	Male	27	Left
9	Male	23	Right
10	Male	26	Right
11	Male	24	Right
12	Female	24	Right

## Data Availability

The data presented in this study are available on request from the corresponding author.

## Supplementary Materials

The following supporting information can be downloaded at: https://www.mdpi.com/article/10.3390/s24010045/s1, Table S1: Classification accuracies and *p*-values of statistically significant brain regions (Congruent vs. Baseline, 0–1 s), Table S2: Classification accuracies and *p*-values of statistically significant brain regions (Congruent vs. Baseline, 1–2 s), Table S3: Classification accuracies and *p*-values of statistically significant brain regions (Congruent vs. Baseline, 2–3 s), Table S4: Classification accuracies and *p*-values of statistically significant brain regions (Incongruent vs. Baseline, 0–1 s), Table S5: Classification accuracies and *p*-values of statistically significant brain regions (Incongruent vs. Baseline, 1–2 s), Table S6: Classification accuracies and *p*-values of statistically significant brain regions (Incongruent vs. Baseline, 2–3 s), Table S7: Classification accuracies and *p*-values of statistically significant brain regions (Congruent vs. Incongruent, 0–1 s), Table S8: Classification accuracies and *p*-values of statistically significant brain regions (Congruent vs. Incongruent, 1–2 s), Table S9: Classification accuracies and *p*-values of statistically significant brain regions (Congruent vs. Incongruent, 2–3 s).
