# Supplementary material for: Temporal Electroencephalography Traits Dissociating Tactile Information and Cross-Modal Congruence Effects
Source: Sensors (Basel). 2023 Dec 21;24(1):45. doi: 10.3390/s24010045 (PMC10780639; doi:10.3390/s24010045)
Supplement: Supplementary file 1 [file sensors-24-00045-s001.zip › sensors-2743000-supplementary.pdf]

sensors

MDPI

Article

Supplementary Materials: Temporal Electroencephalography Traits Dissociating Tactile Information and Cross-Modal Congruence Effects

Yusuke Ozawa, Laura Alejandra Martinez-Tejada, and Natsue Yoshimura

Table S1. Classification accuracies and p-values of statistically significant brain regions (Congruent vs. Baseline, 0-1 s). Regions showing significance were marked with * ($p < 0.05$) or ** ($p < 0.01$).

Region		Congruent Accuracy (%)	Baseline Accuracy (%)	p-value	Region		Congruent Accuracy (%)	Baseline Accuracy (%)	p-value
M1	area 4	68.6	48.3	0.001 **	IPL	PFop	69.2	49.7	<0.001 **
S1	area 1	68.6	48.3	0.001 **		PFt	69.3	47.6	<0.001 **
	area 2	68.7	48.8	<0.001 **		PF	69.7	48.2	<0.001 **
	area 3a	71.9	51.5	<0.001 **		PFm	69.4	49.1	<0.001 **
	area 3b	68.9	48.9	<0.001 **		PGs	63.2	49.3	0.006
S2	area 43	65.3	53.7	0.015 *		PGi	55.5	49.4	0.054
PL	5L	51.5	48.7	0.526		PGp	59.2	49.0	0.021 *
	5m	61.8	48.8	0.036		TPOJ1	72.1	50.4	<0.001 **
	5mv	55.3	49.1	0.052		TPOJ2	65.3	49.4	<0.001 **
SMA	6ma	53.1	52.4	0.983		TPOJ3	61.1	51.8	0.035 *
	6mp	49.0	50.1	0.939	IPS	IP0	58.1	51.5	0.171
	SFL	55.8	49.3	0.322		IP1	55.4	49.4	0.059
	SCEF	50.1	50.1	0.999		IP2	60.6	48.8	0.002 **
PM	6a	52.8	48.6	0.116		IPS1	55.1	50.3	0.350
	6d	54.3	46.5	0.208		AIP	63.9	50.0	0.030 *
	FEF	58.2	53.4	0.495		LIPd	55.4	50.9	0.352
	55b	60.8	52.2	0.101		LIPv	56.9	48.5	0.065
	PEF	67.4	47.2	<0.001 **		MIP	55.0	52.3	0.529
	6v	67.1	48.2	<0.001 **	SPL	7PC	61.1	49.0	0.002 **
	6r	68.6	50.6	<0.001 **		7AL	52.8	50.7	0.654
ACC	33pr	67.2	48.5	<0.001 **		7AM	57.4	46.0	0.025 *
	24dd	60.6	51.2	0.041 *		7PL	49.0	49.6	0.952
	24dv	48.1	49.6	0.854		7PM	59.4	50.8	0.054
	p24pr	70.7	50.5	0.004 **		VIP	50.1	46.6	0.424
	a24pr	57.9	54.2	0.552	POS	POS1	55.5	51.2	0.212
	p24	53.7	50.1	0.434		POS2	65.0	51.6	0.016 *
	a24	50.8	49.4	0.904		DVT	52.9	49.7	0.614
	p32pr	49.3	47.9	0.759		ProS	52.5	49.9	0.323
	a32pr	46.7	49.5	0.575	Occ	V1	55.5	49.5	0.126
	d32	50.1	49.2	0.849		V2	67.8	49.0	0.003 **
	p32	54.2	52.2	0.836		V3	72.2	49.7	<0.001 **
	s32	52.2	50.0	0.806		V4	55.1	54.0	0.952
	area 25	52.1	52.2	1.000		V6	50.1	52.2	0.785
PCC	d23ab	70.3	49.7	<0.001 **		V8	50.6	49.8	0.954
	v23ab	65.9	50.6	0.019 *		PIT	52.3	49.2	0.554
	23c	52.5	47.8	0.229		FFC	54.4	51.1	0.455
	23d	66.4	50.9	0.004 **		VVC	50.5	49.8	0.874
	RSC	66.4	51.4	0.002 **		VMV1	53.3	49.9	0.645
SMG	Ig	57.8	48.1	0.006 **		VMV2	52.2	50.4	0.772
	OP4	70.4	49.9	<0.001 **		VMV3	52.7	51.7	0.845
	OP2-3	63.9	47.8	0.004 **		V6A	55.7	49.5	0.018
	OP1	66.5	47.6	<0.001 **		V3A	51.8	51.4	0.988
	PFcm	68.6	51.3	<0.001 **		V3B	56.1	45.7	0.011 *
	RI	67.6	51.0	<0.001 **		V3CD	55.3	54.4	0.957
	area 52	59.4	46.8	0.002 **		LO1	61.3	50.6	0.055
	A1	65.3	50.3	0.001 **		LO2	61.4	48.2	0.001 **
	Mbelt	65.8	51.1	0.006 **		LO3	54.4	49.4	0.363
	Lbelt	63.9	51.4	0.009 **		V4t	61.7	52.7	0.002 **
	PSL	71.4	50.1	<0.001 **		MT	67.1	49.2	<0.001 **
	STV	69.3	52.6	0.005 **		MST	65.0	52.3	0.011 *
PC	PCV	51.8	50.6	0.943		FST	65.8	49.0	<0.001 **
	7m	63.3	50.0	0.012 *		PH	67.5	47.8	<0.001 **

Table S2. Classification accuracies and p-values of statistically significant brain regions (Congruent vs. Baseline, 1-2 s). Regions showing significance were marked with * (p < 0.05) or ** (p < 0.01).

Region		Congruent Accuracy (%)	Baseline Accuracy (%)	p-value	Region		Congruent Accuracy (%)	Baseline Accuracy (%)	p-value
M1	area 4	46.9	50.9	0.422	IPL	PFop	49.0	46.4	0.721
S1	area 1	45.4	49.8	0.296		PFt	47.1	49.2	0.773
	area 2	45.9	48.3	0.864		PF	46.1	51.3	0.342
	area 3a	44.7	51.3	0.075		PFm	47.1	49.3	0.735
	area 3b	45.8	49.4	0.272		PGs	46.0	51.3	0.015 *
S2	area 43	45.7	49.0	0.629		PGi	54.3	47.7	0.063
PL	5L	49.3	51.9	0.584		PGp	47.2	46.8	0.987
	5M	48.8	49.3	0.988		TPOJ1	49.6	50.0	0.987
	5mv	50.5	48.9	0.218		TPOJ2	43.5	52.3	0.074
SMA	6ma	45.0	49.2	0.267		TPOJ3	46.4	49.5	0.656
	6mp	47.6	49.2	0.875	IPS	IP0	48.7	51.3	0.635
	SFL	46.4	52.4	0.130		IP1	49.7	44.8	0.320
	SCEF	47.6	51.2	0.505		IP2	47.9	48.1	0.997
PM	6a	47.4	50.8	0.571		IPS1	48.8	47.4	0.929
	6d	49.9	50.7	0.903		AIP	50.0	49.6	0.993
	FEF	47.5	52.8	0.204		LIPd	47.1	49.6	0.643
	55b	46.5	51.8	0.051		LIPv	52.4	49.9	0.799
	PEF	45.9	49.0	0.595		MIP	50.4	51.7	0.835
	6v	49.6	50.4	0.931	SPL	7PC	52.3	46.9	0.054
	6r	45.1	49.5	0.147		7AL	52.2	49.9	0.713
ACC	33pr	42.5	49.6	0.051		7AM	47.5	51.6	0.307
	24dd	45.6	51.0	0.142		7PL	51.0	50.1	0.941
	24dv	45.0	50.3	0.350		7PM	44.8	49.4	0.087
	p24pr	50.7	53.9	0.638		VIP	51.3	48.6	0.624
	a24pr	44.2	51.4	0.032 *	POS	POS1	53.3	49.5	0.074
	p24	48.9	48.9	0.990		POS2	47.1	51.7	0.010 **
	a24	46.4	48.8	0.662		DVT	47.1	52.8	0.165
	p32pr	48.2	48.1	0.999		ProS	49.4	51.5	0.231
	a32pr	43.2	49.9	0.093	Occ	V1	50.9	50.9	0.998
	d32	46.9	46.4	0.980		V2	48.5	49.6	0.938
	p32	45.7	50.3	0.329		V3	48.2	50.9	0.541
	s32	45.9	50.8	0.104		V4	47.5	50.6	0.564
	area 25	45.9	50.3	0.268		V6	50.4	53.5	0.450
PCC	d23ab	49.9	49.1	0.885		V8	47.4	51.4	0.232
	v23ab	47.4	51.3	0.394		PIT	55.1	52.3	0.222
	23c	47.6	48.9	0.887		FFC	58.4	53.2	0.097
	23d	48.7	49.9	0.912		VVC	49.9	51.1	0.441
	RSC	47.5	53.1	0.083		VMV1	50.3	50.5	0.991
SMG	Ig	42.6	50.6	0.040 *		VMV2	51.4	52.3	0.743
	OP4	45.9	52.1	0.205		VMV3	50.0	50.8	0.658
	OP2-3	46.1	48.3	0.478		V6A	50.1	47.6	0.802
	OP1	49.2	50.8	0.856		V3A	48.8	50.9	0.770
	PFcm	46.5	49.0	0.759		V3B	47.5	50.7	0.481
	RI	45.0	49.2	0.351		V3CD	48.8	49.9	0.943
	area 52	44.4	49.4	0.093		LO1	47.3	48.8	0.737
	A1	48.5	50.1	0.702		LO2	47.3	49.0	0.898
	Mbelt	49.0	49.6	0.976		LO3	51.3	49.8	0.863
	Lbelt	45.7	54.4	0.004 **		V4t	48.5	47.3	0.922
	PSL	49.0	50.3	0.865		MT	46.1	48.6	0.596
	STV	43.3	51.2	0.199		MST	47.4	50.9	0.436
PC	PCV	44.7	50.6	0.133		FST	47.2	54.1	0.051
	7m	48.8	50.7	0.841		PH	45.28	49.9	0.042 *

Table S3. Classification accuracies and p-values of statistically significant brain regions (Congruent vs. Baseline, 2-3 s). Regions showing significance were marked with * (p < 0.05) or ** (p < 0.01).

Region		Congruent Accuracy (%)	Baseline Accuracy (%)	p-value	Region		Congruent Accuracy (%)	Baseline Accuracy (%)	p-value
M1	area 4	48.2	51.3	0.532	IPL	PFop	50.3	51.8	0.693
S1	area 1	45.4	51.1	0.259		PFt	47.5	49.9	0.761
	area 2	49.6	50.1	0.986		PF	48.5	49.3	0.943
	area 3a	48.6	54.9	0.231		PFm	48.9	52.4	0.484
	area 3b	47.6	52.2	0.409		PGs	46.8	47.9	0.907
S2	area 43	49.7	48.9	0.885		PGi	48.8	48.9	0.941
PL	5L	45.7	50.1	0.287		PGp	46.3	48.9	0.283
	5M	48.1	49.1	0.911		TPOJ1	48.3	47.8	0.984
	5mv	51.1	49.9	0.653		TPOJ2	49.0	48.3	0.951
SMA	6ma	51.5	52.8	0.883		TPOJ3	49.6	48.3	0.887
	6mp	49.3	50.4	0.945	IPS	IP0	48.3	52.6	0.333
	SFL	47.4	50.1	0.493		IP1	48.1	49.6	0.579
	SCEF	50.8	47.2	0.399		IP2	45.9	50.9	0.173
PM	6a	46.3	50.5	0.196		IPS1	45.3	48.5	0.556
	6d	46.5	51.7	0.245		AIP	45.9	50.9	0.283
	FEF	45.3	46.9	0.897		LIPd	50.4	49.4	0.915
	55b	45.7	49.2	0.389		LIPv	50.1	48.9	0.880
	PEF	46.9	50.4	0.429		MIP	46.7	49.5	0.327
	6v	47.5	49.7	0.759	SPL	7PC	46.1	48.9	0.620
	6r	47.1	50.8	0.336		7AL	46.3	49.2	0.704
ACC	33pr	45.1	46.7	0.830		7AM	47.8	48.3	0.949
	24dd	48.5	51.6	0.662		7PL	47.1	48.1	0.970
	24dv	48.3	47.2	0.837		7PM	52.1	51.7	0.996
	p24pr	47.5	48.6	0.885		VIP	53.1	50.6	0.563
	a24pr	51.9	50.7	0.895	POS	POS1	49.9	48.8	0.870
	p24	51.4	49.2	0.772		POS2	47.4	47.9	0.973
	a24	48.5	47.3	0.851		DVT	52.4	52.2	0.998
	p32pr	51.4	48.7	0.324		ProS	51.5	51.1	1.000
	a32pr	49.2	49.6	0.991	Occ	V1	50.5	49.5	0.861
	d32	47.1	54.5	0.083		V2	51.1	50.9	0.997
	p32	50.0	53.3	0.527		V3	47.5	45.8	0.548
	s32	49.6	51.9	0.851		V4	51.3	48.9	0.640
	area 25	48.1	50.6	0.334		V6	48.1	51.5	0.420
PCC	d23ab	49.9	50.5	0.887		V8	51.9	51.2	0.927
	v23ab	49.2	52.2	0.601		PIT	52.1	51.3	0.785
	23c	50.9	50.4	0.963		FFC	50.5	50.2	0.870
	23d	47.2	48.7	0.820		VVC	53.3	51.1	0.549
	RSC	49.6	52.6	0.589		VMV1	51.1	50.8	0.899
SMG	Ig	46.3	51.0	0.078		VMV2	51.4	52.2	0.884
	OP4	47.4	49.9	0.771		VMV3	50.5	51.1	0.755
	OP2-3	46.9	49.3	0.756		V6A	47.4	50.8	0.293
	OP1	47.1	50.1	0.661		V3A	49.4	52.9	0.283
	PFcm	45.8	53.3	0.111		V3B	50.3	46.1	0.220
	RI	47.8	50.2	0.760		V3CD	50.0	46.8	0.527
	area 52	51.9	46.5	0.282		LO1	44.0	53.8	0.016 *
	A1	46.1	51.8	0.082		LO2	43.8	48.3	0.300
	Mbelt	46.5	48.5	0.877		LO3	44.1	49.4	0.131
	Lbelt	46.5	48.2	0.854		V4t	49.6	48.9	0.940
	PSL	46.4	50.1	0.434		MT	49.8	46.8	0.621
	STV	46.1	52.4	0.256		MST	46.9	49.5	0.821
PC	PCV	45.3	51.2	0.053		FST	48.6	51.5	0.657
	7m	52.6	48.9	0.451		PH	45.6	48.4	0.509

Table S4. Classification accuracies and p-values of statistically significant brain regions (Incongruent vs. Baseline, 0-1 s). Regions showing significance were marked with * (p < 0.05) or ** (p < 0.01).

Region		Incongruent Accuracy (%)	Baseline Accuracy (%)	p-value	Region		Incongruent Accuracy (%)	Baseline Accuracy (%)	p-value
M1	area 4	68.8	47.7	<0.001 **	IPL	PFop	72.9	49.7	<0.001 **
S1	area 1	70.6	48.3	<0.001 **		PFt	71.4	47.6	<0.001 **
	area 2	70.4	48.8	<0.001 **		PF	69.9	48.2	<0.001 **
	area 3a	70.8	51.5	<0.001 **		PFm	72.4	49.1	<0.001 **
	area 3b	69.4	48.9	<0.001 **		PGs	63.8	49.3	0.011 *
S2	area 43	70.4	53.7	0.002		PGi	53.3	49.4	0.253
PL	5L	58.6	48.7	0.008		PGp	59.0	49.0	0.025 *
	5m	59.7	48.8	0.015		TPOJ1	73.9	50.4	<0.001 **
	5mv	55.7	49.1	0.057		TPOJ2	69.0	49.4	<0.001 **
SMA	6ma	55.4	52.4	0.705		TPOJ3	68.1	51.8	0.004 **
	6mp	55.9	50.1	0.318	IPS	IP0	59.0	51.4	0.097
	SFL	62.1	49.3	0.031 *		IP1	62.8	49.4	0.001 **
	SCEF	56.4	50.1	0.018 *		IP2	65.8	49.9	<0.001 **
PM	6a	58.9	48.6	<0.001 **		IPS1	57.1	50.3	0.120
	6d	58.0	46.5	0.038		AIP	63.3	48.8	<0.001 **
	FEF	59.0	53.4	0.058		LIPd	60.3	50.9	0.025 *
	55b	67.1	52.2	0.002 **		LIPv	60.1	48.5	0.001 **
	PEF	70.4	47.2	<0.001 **		MIP	58.5	52.3	0.207
	6v	70.6	48.2	<0.001 **	SPL	7PC	61.9	49.0	<0.001 **
	6r	71.3	50.2	<0.001 **		7AL	55.0	50.7	0.173
ACC	33pr	67.2	48.5	<0.001 **		7AM	58.5	46.0	0.013 *
	24dd	64.7	51.2	<0.001 **		7PL	54.6	49.6	0.169
	24dv	55.9	49.6	0.069		7PM	64.7	50.8	0.004 **
	p24pr	70.8	50.5	0.001 **		VIP	55.0	46.6	0.047 *
	a24pr	64.3	54.2	0.015 *	POS	POS1	56.2	51.2	0.062
	a24	57.4	50.1	0.005 **		POS2	67.5	51.6	0.003 **
	p24	57.4	50.1	0.005 **		DVT	52.6	49.7	0.553
	p32pr	52.1	47.9	0.344		ProS	51.5	49.9	0.456
	a32pr	56.7	49.5	0.074	Occ	V1	54.9	49.5	0.321
	d32	54.3	49.2	0.038 *		V2	70.7	49.0	0.001 **
	p32	55.0	52.2	0.507		V3	69.2	49.7	<0.001 **
	s32	54.2	50.0	0.282		V4	61.3	54.0	0.087
	area 25	58.3	52.2	0.329		V6	55.7	52.2	0.615
PCC	d23ab	70.6	49.7	<0.001 **		V8	53.5	49.8	0.343
	v23ab	71.7	50.6	0.001		PIT	53.3	49.2	0.129
	23c	55.0	47.8	0.056		FFC	54.1	51.1	0.085
	23d	68.5	50.9	0.002 **		VVC	51.3	49.8	0.675
	RSC	72.5	51.4	<0.001 **		VMV1	52.8	49.9	0.127
SMG	Ig	63.2	48.1	<0.001 **		VMV2	54.4	50.4	0.097
	OP4	72.9	49.9	<0.001 **		VMV3	53.5	51.7	0.174
	OP2-3	65.0	47.8	<0.001 **		V6A	59.8	49.5	0.020 *
	OP1	68.8	47.6	<0.001 **		V3A	55.3	51.4	0.049 *
	PFcm	67.5	51.3	0.051		V3B	58.6	45.7	0.003 **
	RI	66.5	51.0	<0.001 **		V3CD	57.6	54.4	0.687
	area 52	61.9	46.8	<0.001 **		LO1	65.3	50.6	0.004 **
	A1	57.1	50.3	<0.001 **		LO2	62.2	48.2	0.002 **
	Mbelt	67.5	51.1	0.005 **		LO3	58.3	49.4	0.015 *
	Lbelt	67.4	51.4	<0.001 **		V4t	64.6	52.7	0.005 **
	PSL	70.0	50.1	0.001 **		MT	67.8	49.2	<0.001 **
	STV	69.7	52.6	<0.001 **		MST	62.1	52.3	0.021 *
PC	PCV	58.6	50.6	0.115		FST	65.8	49.0	<0.001 **
	7m	55.6	50.0	0.085		PH	69.9	47.8	<0.001 **

Table S5. Classification accuracies and p-values of statistically significant brain regions (Incongruent vs. Baseline, 1-2 s). Regions showing significance were marked with * (p < 0.05) or ** (p < 0.01).

Region		Incongruent Accuracy (%)	Baseline Accuracy (%)	p-value	Region		Incongruent Accuracy (%)	Baseline Accuracy (%)	p-value
M1	area 4	51.4	50.9	0.982	IPL	PFop	59.2	46.4	0.003 **
S1	area 1	52.4	49.8	0.055		PFt	55.1	49.2	0.265
	area 2	55.8	48.3	0.019 *		PF	52.4	51.3	0.958
	area 3a	54.9	51.3	0.642		PFm	55.1	49.3	0.118
	area 3b	52.1	49.4	0.685		PGs	51.4	51.3	0.999
S2	area 43	52.2	49.0	0.635		PGi	55.5	47.7	0.063
PL	5L	52.8	51.9	0.937		PGp	53.5	46.8	0.137
	5M	53.5	49.3	0.548		TPOJ1	55.3	50.0	0.085
	5mv	52.5	48.9	0.097		TPOJ2	55.7	52.3	0.638
SMA	6ma	48.1	49.2	0.803		TPOJ3	52.1	49.5	0.706
	6mp	49.4	49.2	0.998	IPS	IP0	51.8	51.3	0.946
	SFL	51.8	52.4	0.978		IP1	56.1	44.8	0.010 **
	SCEF	51.3	51.2	0.999		IP2	52.1	48.3	0.209
PM	6a	51.8	50.8	0.919		IPS1	53.2	47.4	0.242
	6d	50.6	50.7	0.998		AIP	55.9	49.6	0.083
	FEF	51.4	52.8	0.857		LIPd	55.3	49.6	0.109
	55b	50.8	51.8	0.891		LIPv	53.3	49.9	0.598
	PEF	54.2	49.0	0.073		MIP	53.9	51.7	0.695
	6v	52.8	50.4	0.810	SPL	7PC	54.0	46.9	0.056
	6r	52.9	49.5	0.416		7AL	54.4	49.9	0.384
ACC	33pr	54.3	49.7	0.151		7AM	50.6	51.6	0.475
	24dd	55.3	51.0	0.176		7PL	55.1	50.1	0.472
	24dv	50.9	50.3	0.989		7PM	53.1	49.4	0.395
	p24pr	54.7	53.9	0.967		VIP	54.9	48.6	0.053
	a24pr	54.0	51.5	0.469	POS	POS1	50.6	49.5	0.344
	p24	54.7	48.9	0.088		POS2	51.4	51.7	0.988
	a24	51.7	48.8	0.488		DVT	48.9	52.8	0.343
	p32pr	54.3	48.1	0.143		ProS	51.1	51.5	0.895
	a32pr	53.6	49.9	0.387	Occ	V1	54.4	50.9	0.164
	d32	54.2	46.4	0.094		V2	54.6	49.6	0.348
	p32	53.2	50.3	0.663		V3	52.2	50.9	0.927
	s32	52.8	50.8	0.577		V4	53.1	50.6	0.789
	area 25	52.4	50.3	0.654		V6	52.8	53.5	0.926
PCC	d23ab	52.2	49.1	0.431		V8	48.8	51.4	0.523
	v23ab	51.6	51.3	0.995		PIT	51.2	52.3	0.534
	23c	53.8	48.9	0.358		FFC	55.6	53.2	0.421
	23d	54.3	49.9	0.534		VVC	52.3	51.1	0.759
	RSC	54.6	53.1	0.794		VMV1	55.5	50.5	0.076
SMG	Ig	50.9	50.6	0.995		VMV2	53.3	52.3	0.327
	OP4	53.1	52.1	0.971		VMV3	51.0	50.8	0.985
	OP2-3	52.6	48.3	0.290		V6A	57.1	47.7	0.034 *
	OP1	52.6	50.8	0.852		V3A	54.7	50.9	0.092
	PFcm	54.3	49.0	0.117		V3B	55.3	50.7	0.260
	RI	52.8	49.2	0.478		V3CD	51.8	49.9	0.783
	area 52	54.7	49.4	0.436		LO1	52.6	48.8	0.422
	A1	51.5	50.1	0.725		LO2	51.3	49.0	0.647
	Mbelt	54.3	49.6	0.274		LO3	54.4	49.8	0.357
	Lbelt	56.3	54.4	0.822		V4t	55.0	47.3	0.108
	PSL	52.9	50.3	0.678		MT	52.1	48.6	0.457
	STV	51.3	51.2	0.999		MST	50.7	50.9	0.998
PC	PCV	51.8	50.6	0.899		FST	53.9	54.1	0.998
	7m	51.5	50.7	0.979		PH	53.3	49.9	0.555

Table S6. Classification accuracies and p-values of statistically significant brain regions (Incongruent vs. Baseline, 2-3 s). Regions showing significance were marked with * (p < 0.05) or ** (p < 0.01).

Region		Incongruent Accuracy (%)	Baseline Accuracy (%)	p-value	Region		Incongruent Accuracy (%)	Baseline Accuracy (%)	p-value
M1	area 4	55.4	51.3	0.289	IPL	PFop	53.6	51.8	0.612
S1	area 1	54.6	51.1	0.315		PFt	56.3	49.9	0.031 *
	area 2	54.3	50.1	0.222		PF	53.5	49.3	0.394
	area 3a	56.1	54.9	0.865		PFm	55.8	52.4	0.378
	area 3b	54.4	52.2	0.761		PGs	53.8	47.9	0.056
S2	area 43	50.9	48.9	0.741		PGi	52.2	48.9	0.231
PL	5L	51.3	50.1	0.914		PGp	51.1	48.9	0.684
	5M	50.9	49.1	0.871		TPOJ1	53.9	47.8	0.089
	5mv	52.2	49.9	0.231		TPOJ2	56.0	48.3	0.033 *
SMA	6ma	53.2	52.8	0.992		TPOJ3	54.4	48.3	0.274
	6mp	55.3	50.4	0.359	IPS	IP0	51.9	52.6	0.974
	SFL	53.2	50.1	0.637		IP1	51.3	49.6	0.787
	SCEF	53.3	47.2	0.200		IP2	53.5	50.9	0.607
PM	6a	50.8	50.5	0.989		IPS1	53.2	48.5	0.421
	6d	53.1	51.7	0.877		AIP	50.6	50.9	0.995
	FEF	49.0	46.9	0.811		LIPd	51.9	49.4	0.672
	55b	52.5	49.2	0.105		LIPv	56.3	48.9	0.079
	PEF	54.3	50.4	0.448		MIP	53.2	49.5	0.422
	6v	53.8	49.7	0.288	SPL	7PC	55.0	48.9	0.292
	6r	56.1	50.7	0.137		7AL	51.8	49.2	0.648
ACC	33pr	55.3	46.7	0.095		7AM	51.1	48.3	0.361
	24dd	56.3	51.6	0.491		7PL	52.5	48.1	0.385
	24dv	56.4	47.2	0.029 *		7PM	51.4	51.7	0.996
	p24pr	55.6	48.6	0.092		VIP	54.6	50.6	0.287
	a24pr	52.8	50.7	0.521	POS	POS1	52.2	48.8	0.443
	p24	55.3	49.2	0.175		POS2	55.4	47.9	0.029 *
	a24	51.4	47.3	0.175		DVT	55.6	52.2	0.463
	p32pr	53.6	48.7	0.193		ProS	52.2	51.1	0.887
	a32pr	56.1	49.6	0.164	Occ	V1	53.0	49.5	0.556
	d32	54.6	54.5	0.999		V2	56.5	51.0	0.045 *
	p32	50.1	53.2	0.510		V3	53.3	45.7	0.022 *
	s32	53.1	51.9	0.940		V4	54.7	48.9	0.311
	area 25	54.7	50.6	0.209		V6	51.9	51.5	0.990
PCC	d23ab	51.9	50.5	0.455		V8	55.7	51.2	0.191
	v23ab	51.5	51.3	0.995		PIT	54.4	51.3	0.441
	23c	57.4	50.4	0.010 **		FFC	53.9	50.2	0.747
	23d	54.2	48.7	0.089		VVC	55.5	51.1	0.383
	RSC	51.8	52.6	0.965		VMV1	53.3	50.8	0.331
SMG	Ig	51.4	51.0	0.991		VMV2	54.4	52.2	0.443
	OP4	51.7	49.9	0.914		VMV3	55.1	51.1	0.290
	OP2-3	50.3	49.2	0.935		V6A	55.9	50.8	0.201
	OP1	53.2	50.1	0.503		V3A	54.4	52.9	0.823
	PFcm	51.4	53.3	0.778		V3B	59.2	46.1	0.002 **
	RI	53.8	50.2	0.376		V3CD	53.8	46.8	0.004 **
	area 52	54.3	46.5	0.083		LO1	50.7	53.8	0.650
	A1	51.5	51.8	0.992		LO2	53.6	48.3	0.286
	Mbelt	52.5	48.5	0.379		LO3	53.6	49.4	0.401
	Lbelt	47.5	48.2	0.984		V4t	53.6	48.9	0.116
	PSL	50.6	50.1	0.985		MT	51.7	46.8	0.032 *
	STV	50.7	52.4	0.814		MST	58.2	51.5	0.026 *
PC	PCV	52.4	51.2	0.927		FST	58.2	51.5	0.026 *
	7m	55.1	48.9	0.050 *		PH	52.9	48.4	0.284

Table S7. Classification accuracies and p-values of statistically significant brain regions (Congruent vs. Incongruent, 0-1 s). Regions showing significance were marked with * (p < 0.05) or ** (p < 0.01).

Region		Congruent Accuracy (%)	Incongruent Accuracy (%)	p-value	Region		Congruent Accuracy (%)	Incongruent Accuracy (%)	p-value
M1	area 4	67.8	68.8	0.858	IPL	PFop	69.2	72.9	0.183
S1	area 1	68.6	70.6	0.759		PFt	69.3	71.4	0.529
	area 2	68.8	70.4	0.558		PF	69.7	69.9	0.998
	area 3a	71.9	70.8	0.692		PFm	69.4	72.4	0.417
	area 3b	68.9	69.4	0.944		PGs	63.2	63.8	0.960
S2	area 43	65.3	70.4	0.076		PGi	55.5	53.3	0.953
PL	5L	51.5	48.7	0.042 *		PGp	59.2	59.0	0.998
	5M	61.8	59.7	0.821		TPOJ1	72.1	73.9	0.614
	5mv	55.3	55.7	0.921		TPOJ2	65.3	69.0	0.150
SMA	6ma	53.1	55.4	0.613		**TPOJ3**	**61.1**	**68.1**	**0.015** *
	**6mp**	**49.0**	**56.0**	**0.022** *	IPS	IP0	58.1	59.0	0.882
	SFL	55.8	62.1	0.024 *		IP1	55.4	62.8	0.049 *
	SCEF	50.1	56.4	0.025 *		IP2	63.9	65.8	0.729
PM	**6a**	**52.8**	**58.9**	**0.023** *		IPS1	55.1	57.1	0.723
	6d	54.3	57.9	0.448		AIP	60.6	63.3	0.549
	FEF	58.2	59.0	0.951		LIPd	55.4	60.3	0.049 *
	55b	60.8	67.1	0.024 *		LIPv	56.9	60.1	0.275
	PEF	67.4	70.4	0.358		MIP	55.0	58.5	0.386
	6v	67.1	70.6	0.185	SPL	7PC	61.1	61.9	0.900
	6r	68.6	71.3	0.239		7AL	52.8	55.0	0.670
ACC	33pr	67.2	67.2	1.000		7AM	59.4	50.8	0.025 *
	24dd	60.6	64.7	0.169		7PL	49.0	54.6	0.145
	24dv	48.1	60.0	0.033 *		7PM	59.4	64.7	0.025 *
	p24pr	70.7	70.8	0.991		VIP	50.1	55.0	0.234
	**a24pr**	**57.9**	**64.3**	**0.023** *	POS	POS1	55.5	56.2	0.756
	p24	53.8	57.4	0.555		POS2	65.0	67.5	0.500
	a24	50.8	53.1	0.734		DVT	52.9	52.6	0.995
	**p32pr**	**46.7**	**56.7**	**0.019** *		ProS	52.5	51.5	0.659
	**a32pr**	**46.7**	**56.7**	**0.019** *	Occ	V1	55.5	54.9	0.873
	d32	50.1	54.3	0.118		V2	67.8	70.7	0.437
	p32	54.2	55.0	0.945		V3	72.2	69.2	0.505
	s32	52.2	54.2	0.611		V4	59.4	61.3	0.047 *
	area 25	52.1	58.3	0.230		V6	50.1	56.0	0.050 *
PCC	d23ab	70.3	70.3	1.000		V8	50.6	53.5	0.504
	v23ab	66.0	71.7	0.021 *		PIT	52.3	53.3	0.654
	23c	52.5	55.0	0.559		FFC	54.4	54.1	0.984
	23d	66.4	68.5	0.512		VVC	50.5	51.3	0.874
	RSC	69.9	72.5	0.559		VMV1	53.3	52.8	0.869
SMG	Ig	57.8	63.2	0.124		VMV2	52.2	54.4	0.342
	OP4	70.4	72.9	0.381		VMV3	52.7	53.5	0.789
	OP2-3	63.9	65.0	0.908		V6A	55.7	59.9	0.206
	OP1	66.5	68.8	0.424		V3A	51.8	55.3	0.301
	PFcm	68.6	67.5	0.863		V3B	56.1	58.6	0.530
	RI	67.6	66.5	0.870		V3CD	55.3	57.6	0.611
	area 52	59.4	61.9	0.536		LO1	61.3	65.3	0.171
	A1	65.3	67.1	0.822		LO2	61.4	62.2	0.910
	Mbelt	65.8	67.5	0.785		LO3	54.4	58.3	0.275
	Lbelt	6.9	67.4	0.470		V4t	61.7	64.6	0.450
	PSL	71.4	70.0	0.857		MT	67.1	67.8	0.970
	STV	69.3	69.7	0.986		MST	65.0	62.1	0.510
PC	PCV	51.8	58.6	0.190		FST	65.8	65.8	1.000
	7m	63.3	64.1	0.846		PH	67.5	69.9	0.528

Table S8. Classification accuracies and p-values of statistically significant brain regions (Congruent vs. Incongruent, 1-2 s). Regions showing significance were marked with * (p < 0.05) or ** (p < 0.01).

Region		Congruent Accuracy (%)	Incongruent Accuracy (%)	p-value	Region		Congruent Accuracy (%)	Incongruent Accuracy (%)	p-value
M1	area 4	46.9	51.4	0.354	IPL	PFop	49.0	59.2	0.027 *
S1	area 1	45.4	52.4	0.056		PFt	47.1	55.1	0.005 **
	area 2	46.9	55.8	0.006 **		PF	46.1	52.4	0.223
	area 3a	44.7	54.9	0.005 **		PFm	47.1	55.1	0.028 *
	area 3b	45.8	52.1	0.041 *		PGs	45.9	51.4	0.090
S2	area 43	45.7	52.2	0.490		PGi	54.3	55.5	0.755
PL	5L	49.3	52.8	0.404		PGp	47.2	53.5	0.051
	5M	48.8	53.5	0.210		TPOJ1	49.6	55.3	0.143
	5mv	50.5	52.5	0.440		TPOJ2	43.5	55.7	0.010 **
SMA	6ma	45.0	48.1	0.156		TPOJ3	46.4	52.1	0.196
	6mp	47.6	49.4	0.811	IPS	IP0	48.8	51.8	0.624
	SFL	46.4	51.8	0.207		IP1	49.7	56.1	0.006 **
	SCEF	47.6	51.3	0.495		IP2	47.9	52.1	0.261
PM	6a	47.4	51.8	0.222		IPS1	48.8	53.2	0.433
	6d	49.9	50.6	0.966		AIP	50.0	56.0	0.077
	FEF	47.5	51.4	0.528		LIPd	47.1	55.3	0.074
	55b	46.5	50.8	0.441		LIPv	52.4	53.3	0.946
	PEF	46.0	54.2	0.002 **		MIP	50.4	53.9	0.535
	6v	49.6	52.8	0.419	SPL	7PC	52.4	54.0	0.713
	6r	45.1	52.9	0.037 *		7AL	52.2	54.4	0.633
ACC	**33pr**	**42.5**	**54.3**	**0.001 ****		7AM	47.5	50.6	0.455
	**24dd**	**45.6**	**55.3**	**0.001 ****		7PL	50.9	55.1	0.250
	24dv	45.0	50.9	0.076		7PM	44.8	53.1	0.070
	p24pr	50.7	54.7	0.358		VIP	51.3	54.9	0.200
	a24pr	44.2	54.0	0.005 **	POS	POS1	53.3	50.6	0.221
	p24	48.9	54.7	0.061		POS2	47.1	51.4	0.270
	a24	46.4	51.7	0.019 *		DVT	47.1	48.9	0.572
	p32pr	48.2	54.3	0.075		ProS	49.4	51.1	0.555
	**a32pr**	**43.2**	**53.6**	**<0.001 ****	Occ	V1	50.9	54.4	0.419
	d32	46.9	54.2	0.076		V2	48.5	54.7	0.047 *
	p32	45.7	53.2	0.034 *		V3	48.2	52.2	0.416
	s32	46.0	52.3	0.030 *		V4	47.5	53.1	0.167
	area 25	45.9	52.4	0.173		V6	50.4	52.8	0.424
PCC	d23ab	49.9	52.2	0.547		V8	47.4	48.8	0.817
	v23ab	47.4	51.5	0.190		PIT	55.1	51.2	0.192
	23c	47.6	53.8	0.087		FFC	58.4	55.6	0.228
	23d	48.8	54.3	0.031 *		VVC	49.9	52.3	0.381
	RSC	47.5	54.6	0.082		VMV1	50.3	55.5	0.095
SMG	Ig	42.6	51.0	0.038 *		VMV2	51.4	53.3	0.330
	OP4	46.0	53.1	0.083		VMV3	50.0	51.0	0.548
	OP2-3	46.1	52.6	0.045 *		V6A	50.1	57.1	0.014 *
	OP1	49.2	52.6	0.322		V3A	48.8	54.7	0.073
	PFcm	46.5	54.3	0.033 *		V3B	47.5	55.3	0.014 *
	RI	45.0	52.3	0.009 **		V3CD	48.8	51.8	0.610
	area 52	44.4	54.7	0.060		LO1	47.4	52.6	0.021 *
	A1	48.5	51.5	0.332		LO2	47.4	51.3	0.572
	Mbelt	49.0	54.3	0.272		LO3	51.3	54.4	0.505
	**Lbelt**	**45.7**	**56.3**	**0.003 ****		V4t	48.5	52.6	0.019 *
	PSL	49.0	52.9	0.231		MT	46.1	52.1	0.226
	**STV**	**43.3**	**51.3**	**0.003 ****		MST	47.3	50.7	0.417
PC	PCV	44.7	51.8	0.036 *		FST	47.2	53.9	0.115
	7m	48.8	51.5	0.616		PH	47.3	53.3	0.033 *

Table S9. Classification accuracies and p-values of statistically significant brain regions (Congruent vs. Incongruent, 2-3 s). Regions showing significance were marked with * (p < 0.05) or ** (p < 0.01).

Region		Congruent Accuracy (%)	Incongruent Accuracy (%)	p-value	Region		Congruent Accuracy (%)	Incongruent Accuracy (%)	p-value
M1	area 4	48.2	55.4	0.096	IPL	PFop	50.3	53.6	0.438
S1	area 1	45.4	54.6	0.033 *		PFt	47.5	56.3	0.088
	area 2	49.6	54.3	0.357		PF	48.5	53.5	0.111
	area 3a	48.6	56.1	0.115		PFm	48.9	55.8	0.131
	area 3b	47.6	54.4	0.125		PGs	46.8	53.7	0.041 *
S2	area 43	49.7	50.9	0.899		PGi	48.8	52.2	0.090
PL	5L	45.7	51.3	0.134		PGp	46.3	51.1	0.313
	5M	48.1	50.9	0.630		TPOJ1	48.3	53.9	0.016 *
	5mv	51.1	52.2	0.550		TPOJ2	49.0	55.9	0.220
SMA	6ma	51.5	53.2	0.741		TPOJ3	49.6	54.4	0.213
	6mp	49.3	55.3	0.193	IPS	IP0	48.3	51.9	0.480
	SFL	47.4	53.2	0.009 **		IP1	48.1	51.3	0.359
	SCEF	50.8	53.3	0.635		IP2	45.9	53.5	0.061
PM	6a	46.3	50.8	0.224		IPS1	45.3	53.2	0.127
	6d	46.5	53.1	0.056		AIP	45.9	50.6	0.149
	FEF	45.3	49.0	0.516		LIPd	50.4	51.9	0.781
	55b	45.7	52.5	0.120		LIPv	50.1	56.3	0.235
	PEF	46.9	54.3	0.150		MIP	46.7	53.2	0.022 *
	6v	47.5	53.8	0.047 *	SPL	7PC	46.1	55.0	0.007 **
	6r	47.1	56.1	0.011 *		7AL	46.3	51.8	0.134
ACC	33pr	45.1	55.3	0.016 *		7AM	47.8	51.1	0.267
	24dd	48.5	56.3	0.011 *		7PL	47.1	52.5	0.068
	24dv	48.3	54.4	0.095		7PM	52.1	51.4	0.960
	p24pr	47.5	55.6	0.044 *		VIP	53.1	54.6	0.538
	a24pr	51.9	52.8	0.913	POS	POS1	49.9	52.2	0.310
	p24	51.4	55.3	0.457		POS2	47.4	55.4	0.011 *
	a24	48.5	51.4	0.393		DVT	52.4	55.6	0.365
	p32pr	51.4	53.6	0.522		ProS	51.5	52.2	0.665
	a32pr	49.2	56.1	0.052	Occ	V1	50.5	53.0	0.490
	d32	47.1	54.6	0.045 *		V2	51.1	56.5	0.125
	p32	50.0	50.1	0.996		V3	48.5	53.3	0.172
	s32	49.6	53.1	0.460		V4	51.3	54.7	0.617
	**area 25**	**48.1**	**54.7**	**0.002** **		V6	48.1	51.9	0.451
PCC	d23ab	49.9	51.9	0.455		V8	51.9	55.7	0.362
	v23ab	49.2	54.7	0.019 *		PIT	52.1	54.4	0.410
	23c	50.9	57.4	0.042		FFC	50.5	53.9	0.331
	23d	47.2	54.2	0.011 *		VVC	53.3	55.5	0.339
	RSC	49.6	51.8	0.568		VMV1	51.1	53.3	0.292
SMG	Ig	46.3	51.4	0.240		VMV2	51.4	54.4	0.183
	OP4	47.4	51.7	0.305		VMV3	50.5	55.1	0.078
	OP2-3	46.9	50.3	0.316		**V6A**	**47.4**	**56.0**	**0.004** *
	OP1	47.1	53.2	0.116		V3A	49.4	54.4	0.167
	PFcm	45.8	51.4	0.416		V3B	50.3	59.2	0.017
	RI	47.8	53.8	0.081		V3CD	50.0	53.8	0.432
	area 52	51.9	46.5	0.231		LO1	44.0	50.7	0.104
	A1	46.1	51.5	0.227		**LO2**	**43.8**	**53.6**	**<0.001** **
	Mbelt	46.5	52.5	0.109		**LO3**	**44.2**	**53.6**	**0.009** **
	Lbelt	46.5	47.5	0.930		V4t	49.6	53.6	0.143
	PSL	46.4	50.6	0.293		MT	49.9	51.7	0.850
	STV	46.1	50.7	0.263		MST	46.9	50.8	0.533
PC	PCV	45.3	52.4	0.038 *		**FST**	**48.6**	**48.2**	**0.002** **
	7m	52.6	55.1	0.667		PH	45.6	52.9	0.133
